# A critical examination of the main premises of Traditional Chinese Medicine

**DOI:** 10.1007/s00508-020-01625-w

**Published:** 2020-03-20

**Authors:** Michael Eigenschink, Lukas Dearing, Tom E. Dablander, Julian Maier, Harald H. Sitte

**Affiliations:** 1 Institute of Pharmacology, Medical University Vienna, Vienna, Austria; 2 Center for Physiology and Pharmacology, Institute of Pharmacology, Medical University Vienna, Waehringer Straße 13A, 1090 Vienna, Austria

**Keywords:** Qi, Meridians, Acupuncture, Pulse diagnostics, Tongue diagnostics

## Abstract

Traditional Chinese Medicine (TCM) consists of a plethora of therapeutic approaches aiming to both characterize and treat diseases. Its utilization has gained significant popularity in the western world and is even backed by the World Health Organization’s decision to include TCM diagnostic patterns into the new revision of the International Classification of Diseases code, the global standard for diagnostic health information. As these developments and potentially far-reaching decisions can affect modern healthcare systems and daily clinical work as well as wildlife conservation, its underlying factual basis must be critically examined. This article therefore provides an overview of the evidence underlying the basic TCM concepts, such as Qi, meridians, acupuncture, pulse and tongue diagnostics as well as traditional herbal treatments. Moreover, it discusses whether scientific literature on TCM reflects the current standard for evidence-based research, as described in good scientific practice and good clinical practice guidelines. Importantly, misinformation regarding the therapeutic efficacy of animal-derived substances has lead and currently leads to problems with wildlife preservation and animal ethics. Nevertheless, the (re-)discovery of artemisinin more than 50 years ago introduced a novel development in TCM: the commingling of Eastern and Western medicine, the appreciation of both systems. The need for more rigorous approaches, fulfilment of and agreement to current guidelines to achieve high-quality research are of utmost relevance. Thereby, ancient knowledge of herbal species and concoctions may serve as a possible treasure box rather than Pandora’s box.

## Introduction

The origins of Traditional Chinese Medicine (TCM) date back more than 4000 years. A first written compilation of TCM was published as *The Yellow Emperor’s Inner Classic* (*Huangdi Neijing*). This publication served as one of the first dogmatic sources for the application of TCM. The *Huangdi Neijing *comprises two books which contain a number of treatises reflecting on the basic and theoretical principles of TCM, as well as its approach to diagnosis, acupuncture and therapeutic applications. Over the millennia, the *Huangdi Neijing* has been annotated and revised numerous times; furthermore, it has been partly translated into English to make the principles and foundation of TCM available to interested practitioners and healthcare professionals worldwide [1, 2].

A first western account of TCM was published in the eighteenth century, when the East Indian Company brought both physicians and medically trained priests to China [3]; however, the communication between the evolving western medicine and TCM was not exhaustive as an exchange of medicinal concepts rarely took place. Indeed, as noted by Nakayama [4], anatomical studies and surgical operations were relatively uncommon in Chinese medicine because of the Confucian tenets of the sacred body. With the principles of yin and yang, the five elements, the universal energy “qi”, the meridians, the inclusion of environmental factors, such as wind, damp, hot and cold, TCM appears as a philosophy that attempts to integrate mind, body, health and disease prevention by diverse practices.

The main principles of TCM have evolved over thousands of years and TCM practitioners also refer to this vast and longstanding experience as a seal of trust. TCM’s fundament is based on its holistic view, the principle of harmony, individuality, and the prevention and treatment of disease. Following these principles, TCM uses unique diagnostic and therapeutic techniques, such as acupuncture, Tai Chi and Qi Gong as well as a plethora of plant and animal derivatives to restore health and prevent illness.

After World War II, a TCM modernization campaign was set up: the leaders of “New China” put major efforts into founding universities, hospitals and research institutes for the promotion of TCM [5]. The first phase led to a rapid increase in popularity and general importance of TCM, resembling a harbinger for the larger changes that were about to follow. The second phase was heralded in the 1980s by Deng Xiaoping’s implementation of the Chinese economic reform, leading to the consolidation and continuous growth of national networks as well as the beginning of international recognition. Nonetheless, the acme was reached during the third phase: in 2015, You-you Tu was awarded the Nobel prize for the discovery of artemisinin [6]. Furthermore, TCM diagnostic patterns were included into the 11th revision of the International Classification of Diseases^1^, which was accepted by the WHO on 25 May 2019 and will come into effect on 1 January 2022.

We ascertained evidence in the field of TCM on the basis of the following topics: (i) TCM publications, (ii) TCM diagnostics, (iii) meridians and acupuncture, and (iv) TCM remedies. It was our intended goal to examine the most obvious and publicly visible ones.

First, we evaluated the increase in publication rates related to TCM, using data derived from the publicly available database PubMed^2^. Then, we searched for highly cited TCM literature and analyzed their content for logical reasoning and scientific justification. In order to elucidate the quality of published articles we performed a substantive analysis of the 100 most cited TCM publications available on PubMed. By using the software Publish or Perish (Harzing, London, UK) we were able to constrain the publications between 2015 and 2019. Next, we assessed TCM’s most commonly applied diagnostic tools—tongue and pulse diagnostics: it is believed by TCM practitioners that the interior of the human body is connected to its exterior and therefore, tongue and pulse diagnosis serve as reference points to determine pathological changes within the organism. TCM views the human body as a holistic unit where all parts are connected by so-called channels and collaterals, the meridians, in which the vital force Qi is believed to be distributed through the entire body. Qi has its roots in a philosophical theory, first described in *The Analects of Confucius* [7]. The meridian system historically consists of 12 main meridians and, while subjected to many studies, has remained unmodified throughout the last two millennia [8]. The rationale behind acupuncture treatment builds on the philosophical foundations of Qi and the meridians, which has been controversially discussed since its inception. Nevertheless, acupuncture is a widely known and broadly applied TCM method, and therefore we discuss arguments regarding its efficacy, safety and utilization. 

Historically, the therapeutics used in TCM derive from the *Guidelines and details of materia medica* (= Bencao gang mu), a book published during the Ming dynasty by the Chinese herbologist Li Shizhen. The Bencao gang mu describes approximately 11,000 different therapeutics and contains information about 1892 herbal remedies [9, 10]. Whilst mostly focusing on herbal remedies, evaluating their therapeutic potential and risk for pharmacointeractions, we also tried to provide a brief overview about ethical considerations regarding the utilization of therapeutics, deriving from endangered species such as the rhinoceros and the pangolin [11, 12]. The utilization of these products, their inhumane production conditions (e.g. retrieval of bear gall), as well as the violation of the *Convention on International Trade in Endangered Species of Wild Fauna and Flora* have been matters of recent animal welfare debates [13, 14].

The critical analysis of TCM, its historical background and philosophical basis also encouraged us to evaluate our own, western, evidence-based medicine (EBM). This was insofar of importance, as (i) we needed to compare TCM to “our” science-based medicine, (ii) it broadened our view for weaknesses in our own medical system and (iii) led us to ascertain the evolution and development of EBM over time. Historically, Scotsman George Fordyce proposed the combination of evidence and medicine in the middle of the eighteenth century; however, the first clinical studies were conducted in 1747 by another Scottish doctor, James Lind, who examined the use of vitamin C in scurvy in a systematic manner; the Hungarian Ignaz Semmelweis solved the etiology of childbed fever roughly 100 years later [15]. Lind, Semmelweis and others in succession converted empirical observation into clinical studies and logical decision making. The development of EBM has undoubtedly also been propelled by major crises as caused by tetanus-contaminated diphtheria antitoxin serum and the toxic ingredients in Elixir Sulfanilamide in the United States of America [16] and the phocomelia caused by the hypnotic drug thalidomide in Germany and other countries [17]. As a result, rigorous drug testing was implemented as both the American Food and Drug Association and the European Medicines Agency were strengthened in their efforts to prevent harm from medicinal products on their way to the market. This endeavor also strengthened EBM since the drugs under scrutiny have to demonstrate efficacy in their clinical target population. Yet, unresolved issues are still present in EBM; however, its approach and ongoing rigorous evolution is unprecedented and without alternative.

Importantly, similar to the development and evolution that EBM went through, TCM also started a process of change and evolution: this process can be gauged with the increasing amount of clinical studies on TCM therapeutics; studies, however, have yet to meet the modern standards described in good clinical practice (GCP) and good scientific practice (GSP) guidelines. To date, the theoretical foundation laid out by the Taoist Confucian philosophy still remains and is deeply embodied and supported by the Chinese government. This political involvement even leads to the aforementioned implementation of TCM diagnoses into WHO publications [13].

## Quantitative and qualitative analysis of TCM publications

**Fig. 1:**
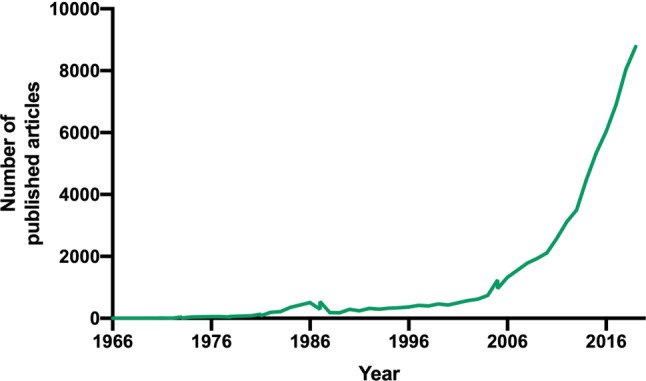
Visualization of publication numbers for the medical subject headings (MeSH) search term Traditional Chinese medicine. The inquiry was conducted on 28.10.2019 using the National Institute for Biotechnology Information´s (NCBI) PubMed database. A timeframe was set, ranging from 1966 to 2019

Information about the quality, quantity, and origin of published TCM articles was examined in the publicly accessible database PubMed: research trend visualizations were performed with the objective of analyzing alterations and developments in TCM publishing frequencies. Accordingly, TCM publication rates have risen almost exponentially throughout the past 30 years, with 8780 published articles in 2019 marking the highest number ever of published TCM articles (Fig. 1).

**Fig. 2:**
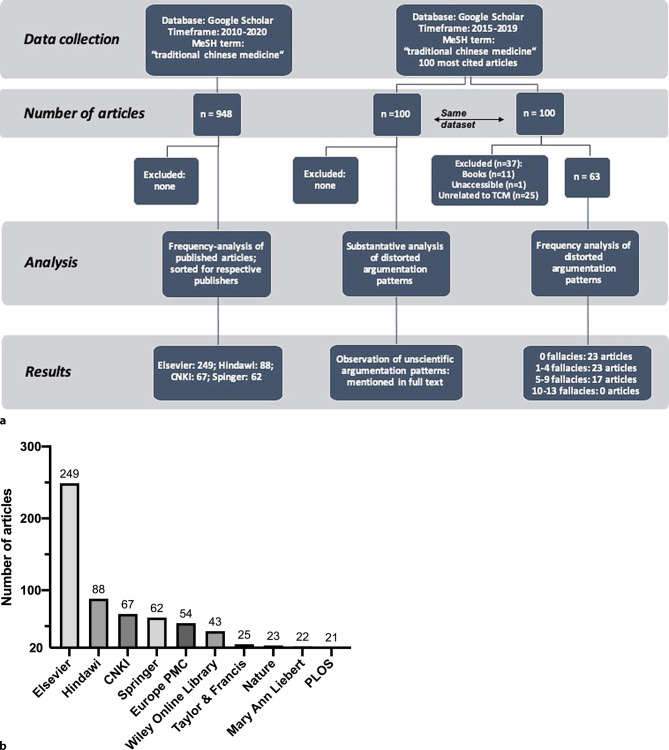
**a** Flow chart demonstrating our approach to quantitative and qualitative analysis of TCM publications. All information was obtained using the program Publish or Perish utilizing the Google Scholar database. The inquiry was conducted on 02.03.2019 and 28.10.2019. **b** Results of the examination of TCM article publishers with more than 20 articles uploaded on Google Scholar. The data were obtained according to the methodology described in (**a**). The absolute numbers have been sorted by the particular publisher and are shown above the bars

Next, we used the software Publish or Perish [113] to obtain 948 articles registered in Google Scholar^3^ including the keyword Traditional Chinese Medicine, published within the timeframe 2010–2020, to identify the publisher with the highest number of TCM articles. We performed a quantitative analysis according to the workflow summarized in Fig. 2a. A total of 101 journals were considered with 11 journals accounting for more than 70% of all published articles, a total of 672 publications. Interestingly, Elsevier had the highest number of published TCM articles in the past 10 years (Fig. 2b). In total, Elsevier, Hindawi, Springer and the China National Knowledge Infrastructure (CNKI) accounted for approximately 50% of all published articles.

**Fig. 3:**
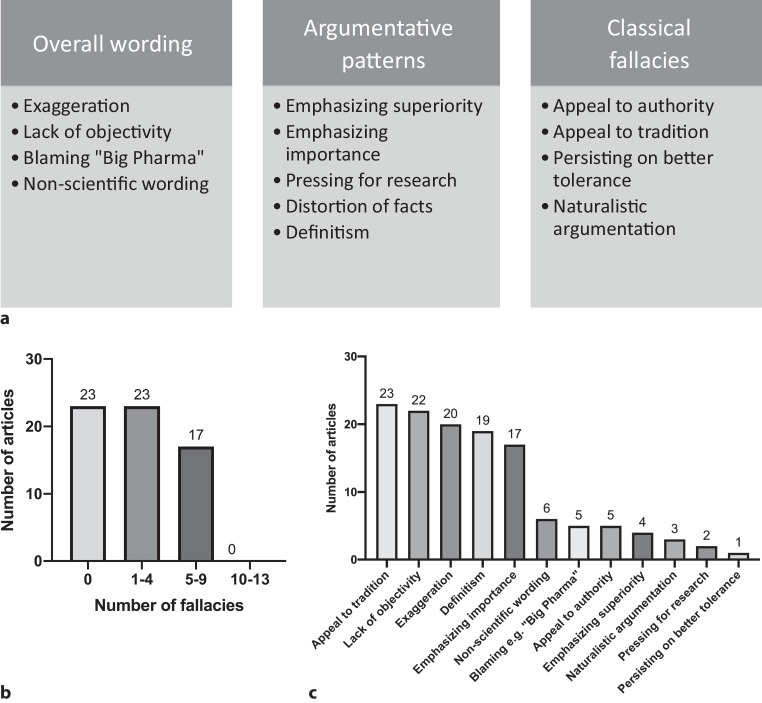
**a** Criteria underlying our predefined binary point system used to evaluate the quality of the 100 most cited TCM publications. If an article met the criteria of one of the sub-areas defined above, one point was assigned to the publication. **b** Results of our frequency analysis of unscientific argumentation patterns. The data were obtained according to the methodology displayed in Fig. 2a. Articles were clustered according to the number of scientific fallacies observed. Each column represents the number of articles included in the respective cluster. **c** Results depicting the frequency of each unscientific argumentation pattern observed in the analysis of the 100 most cited TCM publications. Data were obtained according to the methodology provided in Fig. 2a.

Furthermore, an analysis of the 100 most cited TCM publications registered in Google Scholar^4^ was performed. The data were retrieved similarly to the analysis of journal publication frequencies and adjusted for exclusion criteria (Fig. 2a). Subsequently, the articles were ranked according to a binary point system to ascertain the overall quality of the publications. The point system consisted of three major areas: “overall wording”, “argumentative patterns” and “use of classical fallacies”. As shown in Fig. 3a, these three hypernyms were divided into a total of 13 sub-areas, each resembling a different type of distorted scientific argumentation. If one of these argumentative patterns was used within the title or abstract of an article, 1 point was assigned to the publication—making 13 points the highest possible score. Furthermore, the articles were clustered, forming four different groups. Results are shown in Fig. 3b. Moreover, a detailed frequency analysis of the 13 predetermined argumentation patterns is provided in Fig. 3c.

However, we are aware of the fact that even though our approach was predefined, assigning the points still remains subjective. Because of this limitation the data provided cannot be viewed as a definite, objective examination.

The search was then extended from titles and abstracts to complete pieces of text in order to further elucidate the utilization of unscientific writing patterns. Thereby, several writing patterns frequently found in TCM publications were determined. These patterns mostly resemble argumentative tools used to substantiate statements in an arguably nonscientific way, thus violating the rules of The European Code of Conduct for Research Integrity, published in 2011 by the European Science Foundation^4^. Whilst some authors used naturalistic forms of argumentation such as “herbal remedies are generally better tolerated than synthetic medications” [18], others aided themselves with appeals to tradition or history, e.g. “The Chinese folklore described it as ‘vegetable for long life’ and it has been used for thousands of years in traditional Chinese medicine” [19] or “Since prehistoric times, humans have used natural products, such as plant, […] to alleviate and treat diseases” [20]. A different pattern of reasoning frequently found in TCM publications is the “general usage” argument; e.g. “Traditional medicines, especially herbal or botanic medicines, are very important in health care systems around the world.” [21].

Although some of the aforementioned assertions may sound promising and definite, as well as the fact that discovering and utilizing the medicinal properties of plants will also continue to be an important resource in the future [22, 23], these claims are not substantiated by high quality clinical studies. Approximately 56% of TCM studies uploaded on clinicaltrials.gov constitute a sample size of fewer than 100 subjects or lack statistical power. Furthermore, these studies are prone to statistical type 2 errors [24], therefore less likely to reject a wrong null hypothesis [25]: this imprecision in study design can lead to serious misinformation regarding e.g. clinical endpoints measured in interventional trials.

Despite rising registration rates in ClinicalTrials.gov to more than 10 entries/month and over 570 studies completed (45% of all entries), only 50 TCM studies, a total of 9% have reported results [24]. In contrast, more than 50% of conventional drug trials marked as completed on ClinicalTrials.gov reported results [26, 27]. Such inaccuracies constitute a fertile soil for the usage of unconventional and unscientific argumentation patterns, especially when considering the high risk for type 2 errors and the reporting bias. In general, the methodological quality of clinical trials was reported to be rather low: as can be seen in the literature evaluating the efficacy of TCM medications in the treatment of cardiovascular diseases [28].

Considering nearly exponentially rising publication rates, the need for awareness regarding GSP and GCP is urgent and obvious. Multiple agendas, based on the four elemental concepts of research: honesty, accountability, professional courtesy and fairness, and good stewardship, have been published on the World Conferences of Research Integrity throughout the past years, aiming to provide guidelines for modern state of the art research and dissemination [29]. With respect to clinical trials, GCP guidelines are readily available, regularly updated and even partly implemented in legislation^5^. Many of the aforementioned TCM publications ignore these common scientific conventions that are based on GCP and GSP guidelines. Therefore, they constitute a source for misinformation regarding complex research topics, such as the evaluation of traditional herbal therapies. Moreover, fallacies and unconventional argumentation patterns will continue to undermine the development of more credible, valuable research. An example for underdeveloped research standards is publications attempting to prove the existence of Qi and the meridians.

## Meridians: more than a philosophical concept?

The concept of Qi, the so-called breath or force of life, is a philosophical theory dating back over 3500 years and establishes the basis for most TCM theories [18]. The Qi is believed to flow in certain pathways, so-called meridians, which were first described in the *Huangdi Neijing* [1, 2, 8, 30]. The meridian system historically consists of 12 main meridians and has virtually remained untouched throughout the last two millennia^6^. In order to provide evidence for the existence of meridians, several anatomical, physical and biochemical studies have been conducted; many of which violate the European Code of Conduct for Research Integrity and the aforementioned GSP guidelines.

In an effort to anatomically locate meridians, a French research group led by de Vernejoul applied radiotracers to acupuncture points in a number of separate experiments [31–33]. Thereby, emission patterns were acquired and the authors concluded them to match the classical description of TCM meridians [33]. In addition, the authors reported that only 5% of the applied radiotracer was measurable migrating along blood or lymph vessels [33]. Nevertheless, Guiraud et al. failed to reproduce de Vernejoul’s results [34]. In stark contrast, Guiraud et al. even found the emission patterns to match normal vascular drainage [34]. Furthermore, no difference between radiotracer application on acupuncture points and control points was detectable [34].

A different approach to scientifically validate the existence of meridians relies on changes in electrical conductivity between acupuncture points and control points [35–37]. Publications utilizing this technique often include inaccuracies concerning the way information about the applied methodology is presented. Furthermore, the number of study participants was rather small in most of the publications and the measurements did not conclusively show a difference in impedance in all subjects at all acupuncture points [36, 37].

In addition to the biophysical characterization of meridians and acupuncture points, anatomical and structural equivalents have also long been sought after. Historically, meridians have been described to follow the so-called vascular nerve bundle [38]; however recently, Maurer et al. dissected four cadavers and two lower limbs concluding that meridians and acupuncture points could be related to the fascia corporis externa, muscles, nerves and tendons and to only be merely intertwined with vascular nerve bundles [39]. Therefore, without providing proper evidence for their existence, the authors described an abundance of superficial anatomical structures as a possible anatomical correlate for meridians [39].

Taking publications in these different fields of meridian research into consideration, no scientifically viable explanation for the existence of meridians in a non-philosophical context has been established. Moreover, many studies lack methodological quality and are prone to reporting bias [31–33, 35–37, 39].

## Acupuncture—about popular usage and questionable effect sizes

The use of acupuncture for the treatment of disease is based on the aforementioned rather philosophical, nonscientific concept of Qi and the meridians [40]. Evidence for the use of acupuncture has allegedly been reported as early as 6000 B.C. but remains highly controversial [8]. Acupuncture is widely known and used on a global scale. It is suggested by 90% of American cancer institutes, offered on-site by 70% as a treatment for common chemotherapy side effects [41] and actively asked for by patients as a treatment for migraine [42], musculoskeletal disorders [43] and allergies [44]. Nevertheless, comprehensive reviews containing facts regarding its efficacy, safety and socioeconomic utilization as well as properly conducted clinical studies are barely available, as can be seen from the following examples:

Migraine is the third most common disease in the world [45]. It is a painful burden to many people; time spent in the ictal state is exhausting, making migraine the leading cause of disability in patients with neurological diseases [45]. Although conventional treatment options are widely available, acupuncture is often additionally used to relieve symptoms [46]. A Cochrane review published in 2016 comparing acupuncture therapy to a non-interventional control group, showed moderate evidence for its usage in the reduction of migraine frequency. The authors also concluded that acupuncture was non-inferior to prophylactic drug treatment, however, only in the short-term follow-up setting [42]. Besides that, only small effects of acupuncture over sham treatment were detectable. Despite these seemingly promising assertions, due to an up to 50% risk for attrition and selection bias, the results have to be taken with a grain of salt [42].

With a 4–33% prevalence, lower back pain is the most common musculoskeletal disorder [47]. Even though it was named as the origin for most years lived with disability in 2016 [48], its cause remains unknown in up to 95% of the cases [49]. Therefore, despite receiving conventional pharmacological therapy, patients tend to seek alternatives, such as acupuncture, for symptom relief. A meta-analysis of sham controlled clinical trials showed that acupuncture had a moderate effect over sham acupuncture in pain reduction and no effect on disability reduction [43]. Furthermore, the high levels of heterogeneity observed in the studies included were determined to have originated from sham needle location/depth and insufficient selection of study participants [43].

Allergic rhinitis affects 10–25% of the world’s population [50], causing a significant decrease in work time productivity and overall quality of life [51]. The effect of acupuncture on the severity of symptoms and quality of life in patients with allergic rhinitis and its efficacy in the modern clinical environment largely remains controversial. While some studies reported an effect with possible clinical implications [44], systematic reviews remain inconclusive on whether the reported results are sufficient for acupuncture’s clinical implementation in the therapy of allergic rhinitis [52, 53].

In order to evaluate the biochemical mechanisms underlying acupuncture treatment, an abundance of preclinical studies have been conducted throughout the past centuries [54]. Functional magnetic resonance imaging studies have indicated that acupuncture causes somatosensory, affective and cognitive responses; furthermore, there is moderate evidence for acupuncture’s ability to modulate specific brain areas [55]. Moreover, in the search of molecules viable of transmitting acupuncture’s analgesic effects, adenosine as well as endogenous opioids such as beta-endorphin and dynorphin have been suggested [56]. Adenosine was shown to be released during acupuncture therapy in a mouse model, activating Gi-coupled A1-adenosine receptors, ultimately leading to analgesia [57]. Endogenous opioids were shown to be present in enhanced concentration in the cerebrospinal fluid of patients treated by acupuncture [58]. Furthermore, these findings are supported by the fact that transferring the cerebrospinal fluid of a rabbit that had previously undergone acupuncture to a different, control rabbit led to analgesic effects in this animal; effects that were diminishable by naloxone [59]; however, the experimental data provided in this paragraph only resembles a fragment of the preclinical research performed to elucidate the physiological mechanisms underlying acupuncture treatment. Therefore, it has to be mentioned that critics noted most of the research was done in China, negative results may not have been published and some of the experiments could not be replicated [60].

A frequently occurring issue regarding reviews and meta-analyses about acupuncture is the incoherent control group design of included primary studies. While solutions for the problem of double blinding, such as masked non-penetrating needles exist [61, 62], the validation of effects using sham acupuncture remains questionable [63]. Studies have shown that sham acupuncture effects tend to exceed normal placebo-controlled effects. Moreover, penetrating sham needles obtain the most similar results when compared to conventional acupuncture therapy, therefore reducing effect sizes [64]. Furthermore, evidence suggests that effects of acupuncture may not be specific for the acupuncture point [65, 66].

In order to correctly assume effect sizes, researchers might consider using more than one type of control scenario such as acupuncture point-specific and non-acupuncture point-specific penetrating/non-penetrating control in a double-blinded manner. Nonetheless, conventional acupuncture therapy was shown to be superior compared to all previously mentioned control designs in the treatment of chronic pain [64].

Concerning the safety of acupuncture, only few high-quality data are available. A systematic review published by Chan et al. in 2017 showed analyzed studies lacking in a priori design and sufficient follow-up periods; thus, these studies were not able to screen minor events. Additionally, the majority of the analyzed data were case reports. Even though organ or tissue injuries, infections and local events were reported, severe adverse effects seemed to occur only rarely; however, case control studies ought to be done to further investigate the frequency of adverse effects, determine causes and improve the quality of treatment [67].

Taking the aforementioned considerations into account, acupuncture shows some effect in the treatment of diseases like migraine, lower back pain and allergic rhinitis; however, there is no conclusive scientific basis for its mode of action. Moreover, due to the problem of control group design and the resulting heterogeneity of primary studies included in systematic reviews and meta-analyses, reported effect sizes must be considered with care.

The need for high quality data obtained by carefully conducted studies does not only concern therapeutic approaches like acupuncture. In order to provide an adequate therapy, diagnostic methods have to be thoroughly tested and evaluated too.

## TCM diagnostics—first, check your own pulse

In the clinical use of TCM great emphasis is placed on the principle of diagnostics. The average duration of a TCM consultation is 9 min longer than that of a general practitioner. Therefore, studies indicated that patients treated by TCM practitioners tend to have higher levels of satisfaction [68]. In TCM, a diagnosis is established by combining the principles of inspection, auscultation, olfaction, inquiry and palpation, ultimately creating a so-called pattern diagnosis [69]; however, publications have suggested TCM pattern diagnosis to be subjective to a certain degree: When evaluating its efficacy, the observed level of reliability may be insufficient [70].

In order to provide information about the clinical effectiveness of a diagnostic procedure, scientists usually evaluate its inter-rater and intra-rater reliability. Whilst inter-rater reliability refers to the objectivity of a diagnostic method, intra-rater reliability quantifies its reproducibility.

Multiple studies investigating the accuracy of TCM diagnostics concerning diseases such as lower back pain (LBP) [71], chronic headache [42] and rheumatoid arthritis [72] have been performed. The authors found significant variability in TCM pattern diagnoses and selection of acupuncture points. Moreover, low levels of inter-rater agreement between TCM practitioners were noted. The observed heterogeneity in establishing the right pattern diagnosis and the differences in symptom interpretation pose a challenge to the implementation of standardized therapeutic approaches. In order to propose an adequate therapy, the diagnostic process needs to be rigorously examined. In the following, two diagnostic tools specific to TCM will be highlighted as examples: pulse and tongue diagnosis.

Pulse examination marks a cornerstone of TCM diagnostic principles; it is sometimes even referred to as the most important diagnostic approach in TCM. While the pulse characteristics of a patient may change due to systemic events, such as sepsis or cardiovascular diseases, the utilization of pulse palpation as a viable method for the detection of pregnancy, although described in TCM publications [73], remains highly questionable and is not supported by a broad basis of research. Moreover, the lack of evidence and standardization is also apparent in the pertinent literature concerning TCM diagnostics [74]. Even though recently published articles usually addressed this lack of standardization resulting from the utilization of description patterns that originated more than 500 years ago, viable alternatives for this outdated and rather philosophical categorization are hardly to be found. For instance, pulse is referred to as “… a light knife scraping bamboo, […] a diseased silkworm eating a leaf” [75]. Moreover, articles include questionable statements such as: “tcm doctors are accustomed to assessing pulse by their own perception, rather than on a rational basis” [74].

Additionally, the diagnostic value of pulse examination has been challenged by a study of Walsh et al. in which TCM students were asked to identify basic parameters, such as speed, depth, volume, length and quality of the pulse. The overall agreement level did not differ from that expected by chance alone. Nevertheless, the authors proceeded to conclude that inadequacies in the pertinent literature, the quality of teaching and insufficient clinical practice caused the lack of objectivity, not the reliability of pulse diagnostics itself [76].

In addition to pulse palpation, tongue diagnosis constitutes another hallmark of TCM diagnostics. By evaluating characteristics, such as cracks, color and coating, TCM practitioners try to assess the current health state of, for instance, corresponding internal organs. Even though it seems plausible that a patient’s tongue can exhibit certain characteristics, hinting at either localized diseases, such as oral candidiasis [77] or systemic diseases, such as Sjögren syndrome [78], the evaluation of complex clinical issues is not backed by a broad spectrum of high-quality literature. Furthermore, studies assessing the efficacy of tongue diagnostics found low levels of inter-rater reliability. For instance: a publication by Lo et al. [79] featuring 12 TCM practitioners evaluating a total of 20 patients, found tongue diagnostics to lack inter-rater reliability in key areas: The agreement amongst different observers regarding characteristics, such as tongue color, tongue spots and saliva was reported to be moderate. Moreover, exchange between practitioners did not improve overall agreement [79].

Additionally, in a study conducted by Kim et al. 10 realistic tongue depictions were presented to 30 TCM practitioners in order to assess the subsequent diagnostic consistency. Again, a low inter-rater and intra-rater agreement was found amongst the participants [80].

Research regarding TCM diagnostic methods is still in its infancy. Consequently, the lack of high-quality literature posed a challenge to the evaluation; however, it was apparent that authors who assessed the objectivity and reproducibility of TCM diagnostics unanimously agreed that the principles underlying these examinations need to be standardized in order to fit the modern understanding of medicine. Subsequently, the inclusion of metric data derived from objective biochemical markers is imperative. Moreover, parameters, such as sensitivity, specificity as well as positive and negative predictive values, ought to be evaluated in order to objectify TCM diagnostics and be able to compare their efficacy to western diagnostic methods. Following these thoughts, TCM diagnoses would first have to be objectively and clearly defined.

## TCM therapeutics—forgotten treasure or Pandora’s Box?

The TCM therapeutics historically derive from the “Guidelines and details of materia medica” (= Bencao gang mu), a book published during the Ming dynasty by Chinese herbologist Li Shizhen. The Bencao gang mu describes approximately 11100 different therapeutics and provides information about 1892 herbal remedies [9, 10]. Nowadays, information about TCM medicinal products are included in the “Chinese Pharmacopoeia” published in 2015 [81]: it was established in 1950 by the Chinese Health Ministry as an official document that is now listed on the WHO “Index of Pharmacopoeias” [82]. It comprises a total of 5608 medicinal products and covers more than 90% of TCM medications, including herbal remedies, animal-derived products, minerals, food and conventional drugs^7^. Henceforth, the Chinese government declared the development, utilization and international promotion of TCM as one of their eight steps towards a healthier China^8^.

Regarding the usage frequency of TCM within China, it is estimated that more than 100 million people use traditional medicine every year [83]. This popularity is also met by a continuous increase in the demand for rural areas to cultivate herbs used in TCM. In China alone, it is believed that this demand will rise from 2.3 million hectares of land to an estimated 4.4 million hectares in 2025^9^ [84]. The TCM products are mainly exported to Asian countries, the USA and Europe; however, China also imports enormous amounts of herbs, predominantly from Asian countries^9^ [85]. Similarly, this continuous growth can also be observed with increased demand for animal products. These circumstances pose a great challenge to wildlife preservation and animal ethics^9^. Moreover, the market for products derived from endangered species such as rhinoceros, bear, tiger, lion and pangolin gained popularity in Asia, especially in China, despite persistent trade bans and harsh penalties for their violation since 1993 [11].

Amongst many other therapeutics described in the *Bencao Gangmu*, rhinoceros horn and its derivatives remain one of the most controversial ones. While rhinoceros horn derivatives have officially been excluded from the Chinese pharmacopoeia, studies have indicated that they are still in use for the treatment of medical conditions such as fever, convulsions and pain^10^. This also coincides with reports published by the International Union for Conservation of Nature (IUCN) Species Survival Commission in 2019, indicating China and Vietnam to be the main destinations for rhinoceros horn trafficking^11^.

Despite seizure numbers due to rhinoceros horn trafficking still rising throughout the past 10 years, the answer to the question on where this illegal trade takes place remains somewhat enigmatic; however, since evidence does not implicate conventional pharmacies to take part in the trafficking, it is therefore believed that most of the transactions take place on social media platforms and in the dark net^12^.

Apart from the prominently featured rhinoceros, the survival of more extraordinary species such as the pangolin or scaly anteater is also directly affected by TCM. Pangolins are mammals of the order *Pholidota* and represent the most trafficked species in the world. They are directly threatened by extinction because of their use for medical purposes [86]. In contrast to official bans issued on the therapeutic usage of rhinoceros horn and tiger bones, the use of pangolin claws and scales in the treatment of coronary heart disease, myocardial infarction and stroke is officially permitted by the Chinese government and included in the Chinese pharmacopoeia [87]. Moreover, there are approximately more than 200 pharmaceutical companies producing more than 60 different types of pangolin derivatives, prescribed in about 700 Chinese hospitals [87]. Taking seizure reports into consideration, more than one million pangolins are estimated to have been illegally traded within the last 20 years in Asia alone [87]. China was found to be the most common trafficking destination for large quantity shipments of scales and claws between 2010 and 2015^13^. Thus, TCM might be considered one of the biggest contributors to pangolin trafficking and extinction.

Most recently, the Chinese government reacted and issued an official ban on the coverage of pangolin derivatives by the national insurance^14^. Although rigorous actions will go into effect in 2020, a nationwide ban on the trade of pangolins and the initiation of education programs, aiming at alerting people to alternatives to pangolin derivatives, only seem moderately likely [12]. Thus, the survival of the pangolin once more remains uncertain.

In order to cease the utilization of animal-derived products in TCM, several authors have proposed herbs as substitutes^15^ [88]. This proposition has to be considered carefully, as the evidence for the therapeutic efficacy of herbal remedies utilized in TCM remains poor. Furthermore, potential pharmacological interference with conventional drugs may be common and remains poorly investigated. For instance, high quality evidence about the clinical efficacy of *Pannax ginseng, Lycium barbarum *and *Cinnamon*, the three most extensively exported Chinese herbs, is practically non-existent [89–91]. Moreover, a comprehensive report of Cochrane reviews on Chinese herbal medicines (CHM) suggested that only 16% of all included primary studies use adequate sequence generation. Furthermore, only 7% of these randomized controlled trials applied allocation concealment [92]. Additionally, out of 51 Cochrane reviews on CHM, none were able to provide high-quality evidence necessary for the implementation of CHM therapies in a modern clinical environment [92]. Thus, the substitution of animal-derived products with well-studied therapeutic agents seems to be the logical conclusion.

In contrast to the well-studied mono-therapeutics used in state of the art medical practice, CHMs mostly constitute a mixture of different herbs; however, only few studies provide a detailed chemical analysis of the compounds investigated. This in turn leads to unreliable data, as adulterants and contaminants possibly alter both preclinical and clinical endpoints [93]. Moreover, studies conducted in the late 1990s and early 2000s have shown TCM herbal remedies to be frequently contaminated with other pharmaceutical agents, such as phenytoin, glibenclamide or corticosteroids, therefore, giving rise to potentially fatal interactions [94]. A recent publication indicates an increase in purity found in CHMs. Out of 123 inspected samples, approximately 7% were found to be contaminated [95]. Nevertheless, the erroneous preparation of TCM formulae has led to safety concerns regarding quality standards for TCM therapeutics. Mistakes, such as the accidental substitution of *Stephania tetrandra*, a TCM therapeutic commonly known under the name Fang-ji, with *Aristolochia fangchi* have led to severe medical conditions, such as rapid progressive renal failure [96–98] and the development of urothelial carcinoma [99]. More recently, it has been reported that the main ingredient aristolochic acid, which is still widely used, may also be linked to the development of liver cancer [100]. Similarly, neuropathies and encephalopathies observed in Hong Kong originated from the substitution of CHM *Gentiana rigescens* with *Podophyllum emodi*, a plant used for the extraction of cytostatic agent podophyllin [101].

In contrast to the abovementioned cases, a multitude of biochemical and pharmacological studies are available, many of which indicate interactions with proteins relevant for drug metabolism and transport, such as cytochrome P450 enzymes [102] and ATP-binding cassette transporters [103]. There are implications for the interaction of the commonly prescribed ginseng and warfarin, ultimately leading to a reduced warfarin plasma concentration, thus increasing the chance for thromboembolic events [103, 104]. Publications on interactions between CHM and conventional drugs often lack a qualitative study design and thus, information has to be considered with caution [93, 103, 104].

This poses a challenge to clinicians who frequently encounter patients using over the counter CHMs, often in addition to their originally prescribed drug regimen [105]. Moreover, until 2018, the accessibility to information regarding medicinal products used in TCM and their pharmacokinetic properties was poor. In 2018, a first attempt towards making CHM data available for clinicians worldwide was made with the introduction of the *Encyclopedia of Traditional Chinese Medicine* (ETCM), an online database providing information on commonly used CHMs^16^. The ETCM comprises an abundance of data on pharmacokinetic properties and possible herb-drug interferences, e.g. cytochrome P450 enzyme interactions [106]. Nonetheless, considering the low number of high-quality clinical studies, information originating from this database has to be considered with care.

## The Nobel Prize for the discovery of artemisinin by You You Tu, a Nobel prize for TCM?

Given these obvious challenges concerning TCM medicinal products and the lack of standardization, high-quality randomized controlled trials and even basic pharmacological characterization, other movements should be emphasized, which may be considered in a more positive light. You You Tu, the discovery of artemisinin and the subsequent Nobel Prize may serve as a perfect manifestation of how eastern and western medicine and natural science systems may converge. The entire history of how artemisinin was discovered can be read in the own words of the Nobel Laureate [6]. The important fact behind the discovery was that the search for the new antimalaria drug started from a herbal knowledge base established by the Chinese ancestors; however, it needs to be highlighted that a drug discovery process was applied and successfully completed: the compound, isolated from an extract of the herb Qinghao, used for more than 2000 years against malaria, was thoroughly tested in animal models, clinical trials and chemically characterized with all necessary structure activity/efficacy studies [6]. Importantly, the initial screening failed to produce positive results. Success was only achieved after taking the historically described procedure for the preparation of Qinghao into account, which yielded an effective and ancient treatment commodity for Plasmodium infections for centuries. The promising observations made in these initial studies received highly positive and enthusiastic response on a global scale. This success was the result of an opening of Chinese research to western natural scientific approaches, materialized in form of the Institute of Chinese Materia Medica which—since 1983—was the designated place “for professionals with a modern (western) medicine training background” [6]. Consequently, You You Tu received the Lasker DeBakey Clinical Research Award in 2011 and the Nobel Prize for Medicine or Physiology in 2015 for her outstanding contribution as a scientist. It is also safe to conclude that the discovery of artemisinin and its derivatives can be viewed as a highlight of pharmacognosy, pharmacy and pharmacology; however, some authors of TCM literature utilized this breakthrough discovery to justify their own, in part non-scientific argumentation: “The award of the 2015 Nobel Prize in Physiology or Medicine […] brought the greatest pride and optimism to the natural product community worldwide. […], we are surely entering a New Golden Age of natural products drug discovery” [107]. This statement can only be hoped to be a positive indicator for future directions and that the example of the artemisinin process is exemplary for follow-up studies and developments. Meanwhile, artemisinin and its derivatives have also been found to possess anticancer activity, meaning they are on the stairways to the clinics also in areas other than mosquito-borne infectious diseases [108]. Furthermore, TCM has also been explored in rheumatoid arthritis and some effects were considered to be worthy of further exploration [109]: the Chinese herb formulation Du-Huo-Ji-Sheng-Tang was found to improve lymphatic dysfunction in a transgenic mouse model over-expressing the abundant inflammatory cytokine TNF‑ɑ [110]. Ferulic acid has subsequently been determined as its most active ingredient and shown promising pharmacodynamic effects in lymph vessel drainage and contraction [111].

## Conclusion

The utilization of TCM is based on a vast history dating back thousands of years, thereby justifying the word “traditional” in its title. It is well known in central Europe and its usage, even though hardly backed by scientific studies, enjoys general acceptance amongst the population^17^. This review aimed on examining a number of important areas of TCM covering Qi, meridians, acupuncture, pulse and tongue diagnostics as well as herbal remedies. Furthermore, the publication frequency of TCM literature and the argumentative patterns found in the top cited publications were analyzed. It was observed, that most publications used fallacious descriptions or expressions to substantiate their argumentation. Moreover, it was impossible to determine a scientifically justified basis for the existence of Qi and the meridians; hence, it is difficult to reconcile how acupuncture works as it is based entirely on finding the right acupuncture point; however, there are measurable improvements albeit the evidence can only be graded as mild. It has been speculated that nervous stimulation by short-term pain induction or psychological interference may be the underlying reason for acupuncture effects to occur. Furthermore, an exploration of Tai Chi in comparison to other mobility programs in Parkinson’s disease and other disorders has more recently drawn considerable attention [112]. These TCM-based interventions apparently improve the patient’s ability to move in a reproducible way; however, this cannot be ascertained in many other areas of TCM: The standardization of herbal remedies is difficult to achieve. Moreover, the usage of questionable ingredients deriving of species on the brink to extinction, and the high variability of diagnostic procedures in TCM do not consolidate faith [70]. The transition to science-based diagnostic methods also largely depends on the ability to implement scientific principles, as provided in GSP guidelines, as fundamental parts in the evaluation of diagnostic tools. Furthermore, with TCM diagnosis criteria getting implemented in the 11th revision of the *International Classification of Diseases* which will be actively used by clinicians around the world by January 2022, the safety and efficacy of TCM therapeutics has to be put to the test^18^ [13, 14]. It remains unclear whether this development, which adds traditional ethnomedicine to the armamentarium of clinicians, can be justified over a longer perspective [13]. Furthermore, it needs to be clarified whether social security and healthcare systems will be able to financially cope with the newly implemented “traditions”.

## Footnotes

1 World Health Organization: WHO. ICD-11 International Classification of Diseases in 11th revision. (homepage on the internet; accessed Dec. 02.2019); available from: https://icd.who.int/en

2 PubMED is a database of the National Center for Biotechnology Information (NCBI), a division of the National Library of Medicine (NLM) at the National Institutes of Health (NIH) of the United States of America, accessible via the URL: https://www.ncbi.nlm.nih.gov/

3 https://scholar.google.com

4 European Science Foundation, All European Academies: The European code of conduct for research integrity; 2011 (PDF on the internet; accessed on Dec. 02.2019); available from: https://allea.org/code-of-conduct/

5 Committee for Human Medicinal Products: Guideline for good clinical practice Edition 6; 2016 (PDF on the internet; Accessed on Dec. 02.2019); available from: https://www.ema.europa.eu/en/documents/scientific-guideline/ich-e-6-r2-guideline-good-clinical-practice-step-5_en.pdf

6 WHO Scientific Group on International Acupuncture Nomenclature & World Health Organization: A proposed standard international acupuncture nomenclature: report of a WHO scientific group; 1991 (Homepage/PDF on the internet; accessed on Dec. 02.2019); available from: https://apps.who.int/iris/handle/10665/40001

7 Chinese Pharmacopoeia Commission 京ICP备05016748号: Chinese Pharmacopoeia; [81] (Homepage on the internet; accessed on Dec. 08.2019); available from: http://wp.chp.org.cn/en/content.html?id=ff8080814fd5ba0a014fd6f0402c0143

8 13th five-year plan for economic and social development of the People’s Republic of China.

9 Department of Market Order—Ministry of Commerce: 2017 Chinese herbal medicine circulation market analysis report; 2018 (PDF on the internet; accessed on Dec. 08.2019); available from http://images.mofcom.gov.cn/sczxs/201806/20180626165603110.pdf

10 Natural Resources Defence Council (NRDC), Horizon Key, Kennaugh, A.: Rhino rage: what is driving illegal consumer demand for rhino horn; 2015 (PDF on the internet; accessed on Dec. 08.2019); available from http://www.rhinoresourcecenter.com/index.php?s=1&act=refs&CODE=ref_detail&id=1477908790

11 Milliken T, Emslie RH, Talukdar B.: African and Asian rhinoceroses–status, conservation and trade. A report from the IUCN Species Survival Commission (IUCN/SSC) African and Asian Rhino Specialist Groups and TRAFFIC to the CITES Secretariat pursuant to Resolution Conf (Vol. 9). 2009 (PDF on the internet; accessed on Dec. 08.2019) available from: https://www.cites.org/sites/default/files/eng/cop/15/doc/E15-45-01A.pdf

12 Nowell K.: Species trade and conservation. Rhinoceroses: Assessment of rhino horn as a traditional medicine; 2012 (Pdf on the internet; accessed on Dec. 08.2019); available from: http://www.rhinoresourcecenter.com/index.php?s=1&act=refs&CODE=ref_detail&id=1389669784

13 Heinrich, S., Wittman, T.A., Ross, J.V., Shepherd, C.R., Challender, D.W.S., and Cassey, P.:The Global Trafficking of Pangolins: A comprehensive summary of seizures and trafficking routes from 2010–2015; 2015 (PDF on the internet; accessed on Dec. 08.2019); available from: https://www.traffic.org/publications/reports/the-global-trafficking-of-pangolins/

14 National Healthcare Security Administration: Statement on the insurance coverage of medicinal products; 2019 (Homepage/PDF on the internet; accessed on Dec. 08.2019) available from: http://www.nhsa.gov.cn/art/2019/8/20/art_37_1666.html

15 Bell CM, Simmonds MS, Appiah SS, Howes MJ: Plant substances as alternatives for animal products in traditional medicines; 2006 (PDF on the internet; accessed on Dec. 08.2019); available from: https://www.researchgate.net/publication/251845716_Plant_substances_as_alternatives_for_animal_products_in_traditional_medicines

16 http://www.nrc.ac.cn:9090/ETCM/

17 Unpublished data about the usage patterns and popularity of TCM in Austria (survey conducted in 2019).

18 World Health Organization: ICD-11 Implementation or Transition Guide, Geneva; 2019 (PDF from the internet; accessed on Dec. 08.2019); available from: https://icd.who.int/docs/ICD-11%20Implementation%20or%20Transition%20Guide_v105.pdf
